# A reply to the commentary on ”Animal models of chronic tympanic membrane perforation: in response to plasminogen initiates and potentiates the healing of acute and chronic tympanic membrane perforations in mice” by Wang AY, Shen Y, Wang JT, Eikelboom RH and Dilley RJ; Clin Translat Med, 2014; 3:5

**DOI:** 10.1186/s40169-014-0044-z

**Published:** 2015-02-26

**Authors:** Sten Hellström, Yue Shen, Tor Ny

**Affiliations:** Department of CLINTEC/Otorhinolaryngology, Karolinska Medical University, Stockholm, Sweden; Department of Pathology and Laboratory Medicine, University of British Columbia, Vancouver, British Columbia Canada; Centre for Heart Lung Innovation, St. Paul’s Hospital, University of British Columbia, Vancouver, British Columbia Canada; Department of Medical Biochemistry and Biophysics, Umeå University, Umeå, Sweden

## Correspondence/Findings

A common clinical definition of a chronic tympanic membrane perforation (TMP) is a perforation, which is still open after 3 months. Why the healing of a chronic TMP is arrested is still an enigma. Attempts to generate an animal model for studying the healing pattern of a chronic TMP have until recently been disappointing. However, several studies by the Umeå group, Ny T, Hellström S, Li J, Shen Y and collaborators [1-4] have shown that chronic TMPs can be evoked in a mouse model (plg^−/−^) lacking plasminogen.

In the Commentary by Wang et al. [5] the plg^−/−^ model is questioned and data interpreted such as it in fact may represent an acute TMP model. They claim that the healing studies do not illustrate chronicity of the TMP and that morphologic evidence, such as otoscopic and histological images, is lacking. The authors are supporting their suggestions by adding a solid reference list including our plasminogen reports.

Unfortunately Wang et al. have not carefully scrutinized our studies on the plg^−/−^ model. In the initial report on the mice model [1] the TMPs were followed for 143 days, thus more than 3 months. Furthermore the TMPs were analysed both otomicroscopically and histologically. Otomicroscopically, it appears that all TMPs were closed at days 72 and 143. However, histology showed that all TMPs were still open (Table one in our initial report [1]), but covered by a thick amorphous tissue on the inner surface of the TM (Figure one: G and H and text p. 514 in our initial report [1]). The morphological analysis of the structure also showed the mucocutaneous junction to be located on the inner medial side of the perforation border, characterizing a chronic TMP.

In our more recent publications [2-4] the plg^−/−^ TMP model has been utilized for numerous studies of the healing pattern. To shorten the time for receiving results, and with the knowledge that plg^−/−^ TMPs never heal, most experiments have been initiated 9 days after creating the chronic TMP. The rational for this is that the biological pattern; neutrophil infiltration, fibrin and necrotic tissue deposition, and retarded keratinocyte migration, is histologically the same at day 9 and day 143 after perforation [1]. In another study [3] we showed that the early inflammatory response in the plg^−/−^ model is not altered compared to that in wildtype mice. The chronicity of the perforation in the longterm healing experiment can therefore not be explained by an impairment of the early inflammatory response but rather by an impairment in activation of the inflammatory cells [3].

In conclusion, in contrast to the authors of the Commentary, we have shown, in our publications, that this plg^−/−^ model is a ”true” chronic TMP model. Furthermore our reports show that chronic TMP perforations will heal when treated with plg either systemically, by local injections or topically applied. These findings will be the rational for the clinical studies on chronic TMPs in humans, which are in progress.
